# Perspective: Using Bronchiectasis Action Management Plans for Children With Bronchiectasis—Can It Improve Clinical Care?

**DOI:** 10.3389/fped.2019.00428

**Published:** 2019-10-30

**Authors:** Kobi L. Schutz, Julie M. Marchant, Anne B. Chang, Catherine Turner, Mark D. Chatfield, Gabrielle B. McCallum

**Affiliations:** ^1^Child Health Division, Menzies School of Health Research, Darwin, NT, Australia; ^2^College of Nursing and Midwifery, Charles Darwin University, Darwin, NT, Australia; ^3^Department of Respiratory and Sleep Medicine, Queensland Children's Hospital, Center for Children's Health Research, Queensland University of Technology, Brisbane, QLD, Australia; ^4^Centre for Health Services Research, The University of Queensland, Woolloongabba, QLD, Australia

**Keywords:** children, bronchiectasis, management plan, action plan, randomized controlled trials, clinical trials

## Abstract

While once thought to be rare, bronchiectasis has been increasing globally over the last 15 years. Bronchiectasis is a major contributor to chronic lung morbidity and mortality but remains a neglected disease in respiratory health globally. Currently, few high-level evidence-based management strategies are available for children with bronchiectasis. Strategies to improve clinical outcomes associated with exacerbations are important. In other respiratory conditions such as asthma and chronic obstructive pulmonary disease, use of personalized written management plans have been shown to improve clinical outcomes. Personalized management plans have also been recommended as part of treatment plans in adults with bronchiectasis. We thus undertook a review of the current literature to determine available evidence, and to establish whether a personalized written bronchiectasis action management plan (BAMP) improves clinical outcomes in children with bronchiectasis. Our search identified 43 articles; 16 duplicates were removed and a further 23 were excluded on titles and abstracts alone. Four full-text articles were reviewed but excluded. In the absence of any published studies, it remains unknown whether the use of BAMP is beneficial for improving clinical outcomes for children with bronchiectasis. These results have highlighted this clinical gap and identified the need for high-quality research to inform practice. Until high-quality evidence is available, clinicians are advised to adhere to current national and/or international guidelines.

## Introduction

Bronchiectasis is a chronic condition of the airways characterized by abnormal dilatation of the bronchi and clinically by recurrent or persistent chronic productive or wet cough with episodes of acute exacerbations (1). There are multiple etiologies associated with bronchiectasis, such as congenital malformation, cystic fibrosis (CF) and immune deficiency (2). In some settings, such as socially-disadvantaged populations, recurrent acute lower respiratory infections (ALRIs) (i.e., post-infections) and/or idiopathic etiologies are the most common attributed cause (2). Bronchiectasis is a major contributor to chronic lung morbidity (1) and mortality (3, 4) with the largest reported prevalence among Indigenous populations of high-income countries (1 in 63–68) (5, 6) and in low-middle income countries (2). Despite this burden of disease, there are limited published high-level evidence-based management strategies.

Currently, it is advocated that children with bronchiectasis are managed in accordance to published guidelines (7) so as to improve symptom control, reduce exacerbation frequency, preserve lung function and improve quality of life (QoL) (7, 8). Strategies to improve short- and long-term clinical outcomes associated with exacerbations are important and needed in the management of bronchiectasis, as exacerbations not only adversely impacts the child, but also adds to family stress and family burden (9). Also, severe exacerbations are associated with lung function decline (10). Thus, identifying effective and feasible evidence-based strategies that can reduce the duration and burden of exacerbations and/or improve clinical outcomes for children with bronchiectasis would be beneficial.

One such strategy is to use a personalized, written management plan. These plans are a low-cost resource, designed for patients (or their families) to take home and use as an aid for recognizing signs and symptoms, guide when changes in management are needed and when to seek further medical advice (11). Such plans are used to assist in the home-management of chronic diseases, such as asthma (12, 13) and chronic obstructive pulmonary disease (COPD) (11). For example in people with asthma, personalized written asthma action plans are recommended in all settings, as there is robust evidence that its use improves clinical outcomes [reducing acute doctor visits, respiratory-related hospitalizations (12, 13)] and improves QoL (13). For children with bronchiectasis, standard care usually involves a verbal summary of the management plan with families, with or without a summary letter being sent to the primary treating physician (i.e., general practitioners in primary health care). Indeed, the literature on adults with bronchiectasis identified the need for more information outside of the specialist setting i.e., educational resources to improve knowledge and facilitate better management of their conditions (14).

We propose that the use of personalized action management plans would also improve clinical outcomes for children with bronchiectasis. We undertook a literature search to evaluate the efficacy of a personalized written bronchiectasis action management plan (BAMP) for improving clinical outcomes for children with bronchiectasis. It was anticipated that the routine use of a BAMP may improve clinical outcomes (e.g., reducing respiratory-related hospitalizations and/or improving QoL) through:

1. Improved and earlier identification of signs and symptoms and recommended treatment options leading to better self-management of symptoms, thus halting progression and avoiding urgent doctor visits.
2. Improved communication of management plans between health service providers and patients, e.g., use of type of antibiotics, when hospitalization may be required and results of the most recent bacteria cultured in the patient's airway specimen.
3. Improving self-esteem and/or QoL through better self/parent management.
4. Providing a written reminder when the annual influenza vaccine is due.

### Why Is It Important to Look at This Issue?

Bronchiectasis remains a neglected disease in respiratory health globally (2, 15) and contributes to ongoing morbidity and mortality in children (1). It is also increasingly appreciated that progression of mild bronchiectasis can be halted and/or reversed if treatment is early and optimized (16). Thus, improving clinical outcomes for children with bronchiectasis is important. It is however not yet known whether a personalized written BAMP is effective as written action plans are for children with asthma. Further, while providing each child with a personalized BAMP may be beneficial, its routine use during a clinical consultation adds additional time spent which is an issue as doctors are increasingly asked to do more with less available clinical time. Therefore, it is important to determine the efficacy of using BAMP before it can be successfully implemented in routine clinical care.

## Literature Search and Analysis

### Search Strategy

We conducted a literature search on the 19th July 2019 and planned to include all observational studies and Randomized control trials (RCT) using parellel group design that compared the use of a BAMP vs. a control group (non-use or usual care) for children with bronchiectasis. Children were eligible for inclusion if they were aged <19-years and had a high-resolution computed tomography diagnosis of bronchiectasis. Children were excluded if their bronchiectasis was related to CF or interstitial lung disease.

Our primary outcome measures were the number of participants who had one or more exacerbation over the study period, rate of exacerbations hospitalization over the study period. Secondary outcome measures were symptom control (dyspnea, cough, wheeze), QoL scores [e.g., bronchiectasis QoL, PC-QoL (17)], pulmonary lung function tests indices [e.g., FEV_1_, FVC], functional status [e.g., 6-min walking test, sit to stand test (18)] and mortality.

Databases were searched from inception until our search date (19th July 2019) and articles were restricted to English. We also searched the reference list of full-text articles for any additional studies. See Supplementary Material for search strategy. The search was conducted using the following databases:

1. The Cochrane Central Register of Controlled Trials (CENTRAL)
2. The Cochrane Airways Group Specialized Register;
3. MEDLINE (PubMed)
4. ClinicalTrials.gov
5. World Health Organization International Clinical Trials Registry platform trials portal (WHO ICTRP)
6. Australian and New Zealand Clinical Trials Registry (ANZCTR).

### Analysis

Two authors planned to independently assess risk of bias for each study using the criteria outlined from the Cochrane handbook for systematic reviews, according to the following domains, high, low or unclear (19). We had planned to analyze dichotomous data as odds ratios (ORs), and continuous data as mean differences (MDs) or standardized mean differences (SMDs). For dichotomous data, we had planned to report the proportion of participants contributing to each outcome in comparisons vs. the total number randomized. For cluster RCTs, only data adjusted or for clustering would have been used. We had planned to exclude cross-over trials as such designs are not appropriate for this intervention.

We planned to describe any heterogeneity between study results to determine if this reached statistical significance using Chi^2^. We considered heterogeneity as significant when the *P*-value was < 0.10 (19) and planned to measure heterogeneity among studies in a meta-analysis using the *I*^2^ statistic. If there was substantial heterogeneity, we had planned to explore possible causes using prespecified subgroup analysis (as possible) (19). Subgroup analysis was planned by study setting (high income vs. low-middle income countries) and ethnicity (Indigenous vs. non-Indigenous).

## Results

**Figure 1:**
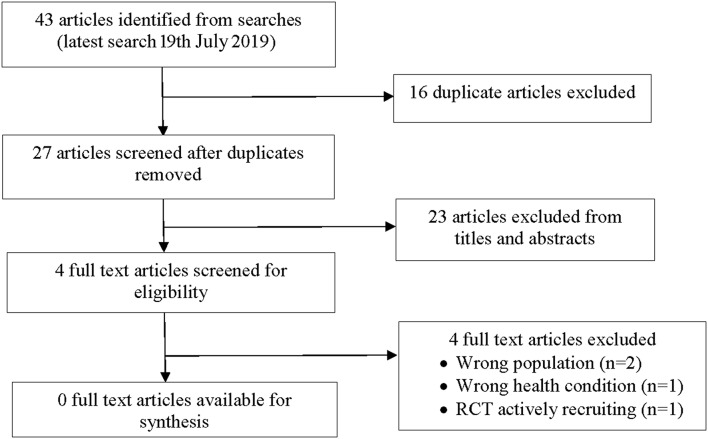
Study flow diagram.

A total 43 potentially relevant articles were identified from our searches (Figure 1). Nine articles were identified from PubMed; 23 articles from the Cochrane Systematic Review database, four articles from the Australian and New Zealand Clinical trials registry, four articles from Clincaltrials.gov and three articles from the World Health Organization trials portal. After 16 duplicates were removed, 27 articles were screened and 23 articles were excluded on title or abstract. Of the 23 articles, four were the wrong population (adult studies), 12 did not include bronchiectasis participants and 7 were the wrong intervention. Four full text articles were reviewed and subsequently excluded with reasons detailed below and in Figure 1. No study (RCT or observational) met the inclusion criteria for this review.

Of the four articles that were excluded, one was a Cochrane Systematic review assessing the efficacy of self-management interventions for non-CF bronchiectasis involving adults (20). There were no studies in children in the Cochrane Systematic review (20) and was thus excluded.

The second article was a Cochrane Systematic review examined the use of caseworker compared to non-caseworker assigned discharge plans for children with chronic respiratory illnesses (i.e., asthma, bronchiectasis) to reduce hospital readmissions (21). We excluded this review as none of the four studies involved children with bronchiectasis.

The third article was an RCT, which examined whether use of a bronchiectasis evaluation tool (BET) compared to standard care increased self-management of bronchiectasis and increased QoL (22). We excluded this article as only adults were included.

The fourth RCT was identified on the Australian and New Zealand Clinical Trials Registry (23). This RCT in children <19-years with either chronic suppurative lung disease or bronchiectasis aims to determine whether the use of a personalized written BAMP vs. usual care from their respiratory physician improves clinical outcomes [reducing non-scheduled doctor visits and cough-specific quality of life (17, 24)]. This study is actively recruiting participants and was thus excluded.

## Discussion

There are currently no publications on the efficacy of a personalized BAMP for improving clinical outcomes in children with bronchiectasis.

In other studies, such as on COPD, self-management plans not only improved QoL but have also reduced respiratory-related hospitalizations (11, 25). In adults with COPD, personalized action plans used over a 12-month period reduced the likelihood hospital admissions (OR = 0.69, 95%CI 0.49,0.97) and emergency department visits (OR = 0.55, 95%CI 0.38, 0.78) (11). In another study in COPD patients, use of a personalized management plan over a 12-month period significantly improved health-related QoL on the St George Respiratory Questionnaire (mean difference−2.69 points; 95%CI−4.49, −0.90) (25). Similar data has also been shown in adult studies with asthma, whereby QoL scores improved participants who received an action asthma plan compared to those who did not receive a plan (MD 0.18, 95%CI 0.05, 0.30) (13). In children with asthma, use of a written asthma action management plan improved QoL scores for in pediatric care givers (*p* ≤ 0.0001) (12).

A Cochrane review (20) recently highlighted the lack of evidence to meaningfully assess the benefit of written BAMP in adults, with only two small poor-quality studies included in review. The authors concluded that further high-quality studies are needed in adults, as well as children (20). Our search further supports this finding of a paucity of research evaluating action management plans for bronchiectasis in children.

In the absence of published studies, we are unable to determine whether the routine use of a written BAMP improves clinical outcomes for children with bronchiectasis. Thus, there is a need for high-quality RCTs to determine the effectiveness of a BAMP that include a comparative group of usual care to assess improvement in clinical outcomes for children with bronchiectasis. Such well-designed RCTs should include important validated outcomes, such cough-related QoL and rate of a-priori defined respiratory exacerbations.

We suggest that future BAMPs consist of several key points: (a) baseline characteristics e.g., details of the child's bronchiectasis (etiology, severity and location, microbiology of the child's latest airway specimen), (b) what the child's daily treatment regimen is (e.g., type of medication(s) and frequency, airway clearance technique), (c) what to do when there is a flare up (including medications), (d) indications when to see a doctor and, (e) details of when their annual influenza vaccine is due. Until high-quality evidence is available, clinicians are advised to adhere to current national and/or international guidelines.

## Data Availability Statement

All datasets for this study are included in the article/Supplementary Material.

## Author Contributions

AC conceptualized the study. GM conceptualized the review. KS and GM independently undertook the search strategy. KS wrote the first draft of the manuscript. GM and AC substantially revised the manuscript. JM, CT, and MC made an intellectual contribution to the review of this manuscript. All authors approved the manuscript.

### Conflict of Interest

The authors declare that the research was conducted in the absence of any commercial or financial relationships that could be construed as a potential conflict of interest. The authors are currently undertaking a RCT evaluating the routine use of BAMP in children.
