# Supplementary material for: Perspective: Using Bronchiectasis Action Management Plans for Children With Bronchiectasis—Can It Improve Clinical Care?
Source: Front Pediatr. 2019 Oct 30;7:428. doi: 10.3389/fped.2019.00428 (PMC6831557; doi:10.3389/fped.2019.00428)
Supplement: Supplementary file 1 [file Table_1.DOCX]

Supplementary Material

# Search Strategies

**MEDLINE (PubMed) (RCT search)**#1 Bronchiectasis
#2  (action plan* OR action management plan* OR management plan* OR plan*)
#3 (child* OR ped* OR paed* OR adolesc*)
#4  (#1 AND #2 AND #3)
#5 (control* clinical trial OR randomi* control* trial)
#6  randomi*
#7 placebo
#8  trial
#9 groups
#10 ((#5 OR #6 OR #7 OR #8 OR #9))
#11 Animals
#12 Humans
#13 (#11 NOT (#11 AND #12))
#14 ((#4 AND #10) NOT #13)

**MEDLINE (PubMed) (Observational study search)**#1 Bronchiectasis
#2 (management plan OR action plan)
#3 (child* OR ped* OR paed* OR adolesc*)
#4 (#1 AND #2 AND #3)

**Cochrane Airways Group Specialized Register and**#1 bronchiectasis: ti,ab,kw
#2 "action management plan*" or "management plan*" or "action plan*"
#3 child* or ped* or paed* or adol*;ti,ab.kw
#4 {and #1-#3}

**Cochrane CENTRAL**#1 bronchiectasis:ti,ab,kw
#2 "action management plan*" or "management plan*" or "action plan*"
#3 child* or ped* or paed* or adol*;ti,ab.kw
#4 {and #1-#3}

**WHO ICTPR / ANZCTR / ClinicalTrials.gov**Bronchiectasis AND (management plan OR action plan)
